# Cdk5: An Emerging Kinase in Pain Signaling

**DOI:** 10.4172/2168-975X.S1-003

**Published:** 2012-10-03

**Authors:** Tej Kumar Pareek, Lisa Zipp, John J Letterio

**Affiliations:** Department of Pediatrics, Division of Pediatric Hematology and Oncology, University Hospitals Case Medical Center, Case Western Reserve University, Cleveland, OH 44106, USA

## Abstract

Pain is an important survival mechanism for an organism. It can turn into severe mental and physical disorder however, if the molecular and/or cellular pathways involved in pain signaling are altered. Chronic pain is characterized by an altered pain perception that includes allodynia (a response to a normally non-noxious stimulus) and hyperalgesia (an exaggerated response to a normally noxious stimulus). Past few years of pain research has been mainly focused on precise understanding of the molecular and cellular nociceptive signatures altered during chronic pain, so that more effective pain relievers can be developed. The importance of protein kinases in normal cellular homeostasis and disease pathogenesis has evolved rapidly in the past few decades. The recent advancement defining the role of multiple protein kinases in regulating neuronal plasticity and pain sensitization has gained enough attention of pharmaceutical industry to develop specific and selective kinase inhibitors as analgesics. Cyclin-dependent kinase 5 (Cdk5) is one such emerging kinase in pain biology. We will discuss here the recent advancement and therapeutic potential of Cdk5 in pain signaling.

## Introduction

Pain is both a highly important health problem and an increasingly mature topic of study. Over one-third of the world's population suffers from persistent or recurrent pain. To an estimate chronic pain affects approximately 116 million American adults alone, more than the total affected by heart disease, cancer, and diabetes combined. Medical conditions including diabetes, AIDS, and multiple sclerosis all have a high incidence of chronic neuropathic pain. Because pain impairs one's ability to carry out a productive life, it has serious economic consequences in addition to being a major health problem. Pain alone costs the nation up to $635 billion each year in medical treatment and lost productivity [1]. It is important to note that despite this mind boggling statistics only <2% of the NIH budget goes to fund pain research. Thus, the 2010 Patient Protection and Affordable Care Act required the Department of Health and Human Services (HHS) to enlist the Institute of Medicine (IOM) in examining pain as a public health problem. Te idea of pain is not new to us and a notable information regarding pain has emerged since 18^th^ century [2]. Since then, scientists have made remarkable strides to understand the biological, cognitive, and psychological underpinnings of pain. However, despite much work and thought, fundamental issues about pain remain unresolved. Notably, these include whether pain results from the activity of a dedicated neural apparatus or is the product of less specific processes. An important focus of pain research has been the study of chronic pain mechanisms, particularly the processes that lead to the spontaneous pain and hyperalgesia associated with these states. The use of currently available pain medication and therapies is limited partly due to their deleterious side effects and inadequate efficacy. For example, morphine and its analogues are the most effective analgesics for treating severe and terminal pain but they are frequently under-prescribed because of the fear that, as tolerance to the drug develops, dependence and addiction will follow. Therefore, it's quite evident that still many gaps persist, and developing more effective and less risky pain relievers remains a major challenge.

To the excitement of pain biologists, the last few decades have been an incredibly productive time in pain research. Information from recent scientific discoveries is virtually exploding and has revealed numerous novel targets for the advent of new pain therapies. Major advances have occurred at levels spanning from molecular studies that have identified transduction proteins in nociceptors to cortical imaging studies which reveal how pain is experienced on a cognitive level [3,4]. Cellular networks involved in perceiving pain transduction and perception complexly involve fundamental biological events at multiple levels of the nervous system. Nociceptors are the first fundamental unit of this cellular network. This subpopulation of primary sensory neurons is activated by different noxious stimuli such as heat, cold, chemical sensation, mechanical sensation, inflammation, etc. Identification of cellular and molecular targets that are altered on specific populations of nociceptors during different types of pain have helped in the development of novel pain therapies that target specific mechanisms on identified populations of nociceptors. Elegant molecular genetic studies conducted in the past few years have now enabled us to identify specific molecules that are involved in the processes of pain transduction. Recently, David Julius group has discussed about these cellular and molecular nociceptive signatures at length [5]. The major advancement in understanding the nociceptive signaling came through the discovery of ion channels in this process [6], specifically vanilloid receptors (VR), also known as transient receptor potential vanilloid (TRPV) channels, involved in detecting temperature, chemical and inflammatory pain [7,8].

Furthermore, recent advancement in molecular biology and completion of the human genome sequence has revealed the significance of protein kinases in maintaining normal cellular homeostasis and multiple disease pathology including their involvement in regulating neuronal plasticity and pain sensitization. Therefore, there is a growing interest in developing protein kinase inhibitors for the treatment of a number of diseases. More than 20 protein kinase inhibitors are already in clinical trials, and many others have entered in either clinical trial without their structure being disclosed or are still in preclinical studies [9]. Protein kinases are becoming the second largest group of drug targets after GPCRs, accounting for 20%-30% of drug discovery activity in many pharmaceutical companies. Although many protein kinase inhibitors are in clinical trials for treating different diseases, especially cancer, they are not specifically being tested for clinical pain. Here we will overview the involvement of protein kinases during pain signaling with specific focus on a newly emerging protein kinase, Cyclin-dependent kinase-5 (Cdk5) in this process.

## Cdk5 and its role in pain signaling

**Figure 1:**
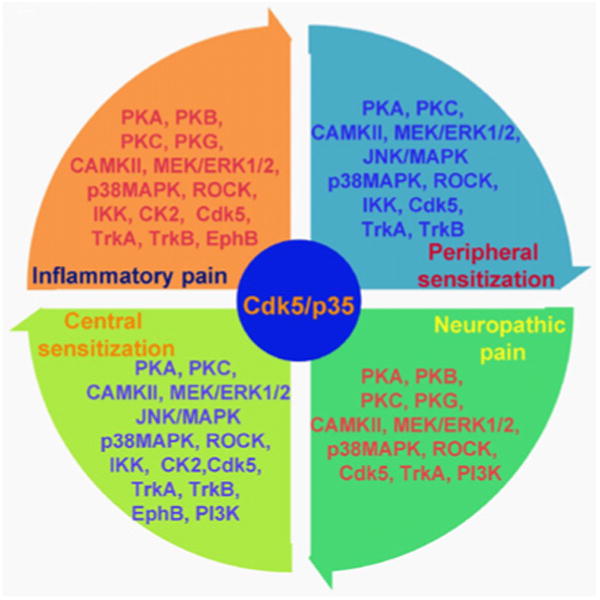
An overview of involvement of multiple protein kinases in different aspects of pain such as, peripheral sensitization, neuropathic pain, central sensitization and inflammatory pain. Cdk5/p35 signaling can directly or indirectly influence the activity of these kinases.

The human genome encodes 518 protein kinases (constituting about 1.7% of all human genes), representing one of the largest protein families [10]. It is quite clear now that functionality of any protein in cell is determined by its post-translational modification, and kinases are the key fundamental enzymes involved in this process. Thus, the involvement and significance of kinases has been reported nearly in every cellular dynamic process including but not limited to metabolism, transcription, cell cycle, cytoskeletal rearrangement, apoptosis, cellular mobility and differentiation. Protein phosphorylation also plays a critical role in intercellular communication during development, in physiological responses, and in the proper functioning of the nervous and immune systems. Mutations and deregulation of protein kinases play causal roles in human disease, affording the possibility of developing agonists and antagonists of these enzymes for use in disease therapy [9,11,12]. Although protein kinases were not favored as targets for analgesics, studies in the last decade in various animal pain models [13] have demonstrated important roles of these kinases in regulating neuronal plasticity and pain sensitization following intense noxious stimuli and injuries. Both serine/threonine and tyrosine kinases have been reported to be involved in multiple aspects of pain signaling (Figure 1). Furthermore, protein kinases also play a central role in the short- and long lasting effects induced by opiates and other abused drugs, participating in the acquisition of tolerance, sensitization, and other behavioral hallmarks of drug addiction [14,15].

## Cdk5 and pain; lessons from pharmacological inhibition and gene targeting

Cdk5 is a unique member of the small proline-directed serine/threonine kinase family. Initially misnamed due to its close sequential homology with other Cyclin-dependent kinase (Cdk) family member Cdk2, this kinase plays a limited role in cell cycle and has restricted association with cyclins. For a long period of time it has been thought that Cdk5 is mainly active in post-mitotic neurons due to the abundant and selective expression of its obligate partner proteins, p35 and p39 in these cells [16,17]. These notions about limited neuronal role and regulation of Cdk5 activity have been challenged however, by recent reports from Herrup et al., reporting its involvement in cell cycle, [18-21] indicating the regulation of Cdk5 by association with cyclin-I [22,23]. Moreover, a series of reports has been published from multiple research labs in the past few years, arguing the importance of this kinase beyond neurons [24-26]. Collectively, our current knowledge about this kinase suggests that in the central nervous system (CNS) normal Cdk5 activity is required for maintaining normal neuronal homeostasis and development whereas deregulated Cdk5 activity leads to neurodegenerative disorders [27-29]. In spite of the ubiquitous presence of Cdk5 protein, its activity in peripheral nervous system (PNS) and non-neuronal tissues is hardly detected under normal conditions. However, the higher expression of Cdk5 and its activity are detected in these tissues under pathological conditions such as inflammation, cancer, diabetes etc., which coincides with upregulated p35 protein levels [24,26].

In 2006, we unraveled for the first time an unprecedented role of Cdk5 in PNS and showed its essential requirement in nociceptive pathway [30]. Since then, these results were further confirmed by several other research groups [31-37]. Essentially now it is well established that both Cdk5 and its activator p35 are present in dorsal root ganglion (DRG) and trigeminal ganglion (TG) and the expression and activity of Cdk5/p35 is increased upon nociceptive stimulation [30]. It has been reported that intrathecal administration of Cdk5 selective inhibitor, roscovitine not only attenuates formalin-induced nociceptive response but also diminishes morphine tolerance [38,39]. Based on these studies, a systemic evaluation of Cdk5 inhibitors on morphine induced analgesia and tolerance has been performed [40]. Analysis of the *in vivo* role of Cdk5 in pain signaling has been restricted due to the embryonic lethality of the Cdk5-knockout mice; however, it should be noted that Cdk5-knockout pups are unresponsive to noxious cutaneous pinch [41]. Moreover, genetically altered mice lacking p35 (p35-/-) or overexpressing p35 transgene (Tgp35) further validated the biological significance of this kinase, showing hypoalgesic and hyperalgesic behavior against heat and inflammatory pain, respectively [30]. As discussed above TRPV channels play an important role in pain signaling. We identified TRPV1 as a direct substrate for Cdk5/p35 kinase [42]. Cdk5-mediated phosphorylation of TRPV1 at threonine-407 can modulate agonist-induced calcium influx, and this effect could be reversed by restoring Cdk5 activity. These results were further confirmed in primary nociceptor-specific Cdk5 conditional-knockout mice. These mice showed reduced TRPV1 phosphorylation, resulting in significant hypoalgesia and establishing the importance of Cdk5-mediated TRPV1 phosphorylation in its functional regulation specific to pain signaling [42]. The life span of these mice is not compromised however; these mice develop skin lesions upon aging due to general sensation loss, as evident from deep skin scratches that turn into unhealed wounds [43]. Further characterization of these mice showed that activity and expression of Cdk5/p35 is restricted to C fibers, and Aβ and Aδ fibers are spared with this kinase activity. This was also reflected in multiple pain testing where these mice showed significant altered response to heat, pain, inflammation and chemical induced hyperalgesia but not to mechanical stimuli.

## Role of Cdk5 in morphine tolerance and opioid addiction

It is known that opioid analgesics are the most efficacious drugs used to relieve severe pain; however, chronic administration of these drugs can lead to the development of tolerance and dependence, processes that are intimately related to opioid addiction. The processes underlying opioid tolerance involve complex compensatory changes in many opioid and non-opioid neuronal circuits [44-46]. However, the molecular mechanisms underlying the development of morphine tolerance remain controversial. A growing body of literature suggests that Cdk5/p35 plays an important role in opioid addiction and morphine tolerance. Both p35-/- (lacking Cdk5 activity≈60%-70% lower than wild type) or Tgp35 (with higher Cdk5 activity≈2-4 fold higher than wild type) mice depict altered response towards morphine tolerance. More rapid development of morphine tolerance in Tgp35 mice suggests that hyperactivation of Cdk5 is an important factor in the development of tolerance [47]. Recently, it has been demonstrated that opioid addiction in humans and in rats is associated with neuronal Cdk5/p35 levels, which cause aberrant hyperphosphorylation of NF-H proteins [48]. Cdk5/p35 and downstream signaling in the ventral striatum play a critical role in the effects of acute METH treatment as well as the development of behavioral METH sensitization [49]. Chronic morphine exposure upregulates the transcription factors cAMP response element binding protein (CREB) and *δ-fosB*, both of which appear to partially mediate an aspect of tolerance [50,51]. *δ-fosB* is thought to be a sustained molecular switch for addiction [51]. Cdk5 is a downstream target gene of *δ-fosB*, and it regulates the effects of chronic cocaine exposure in mice [52,53]. Induction and/or activation of Cdk5 are also implicated in the development of psychological dependence on morphine [54]. As reported above, intrathecal roscovitine administration also prevents the development of morphine tolerance [39].

## Functional regulation and molecular involvement of Cdk5 during pain signaling

It is quite clear that activity of Cdk5 is required to generate a nociceptive response; however, there is some ambiguity around the functional regulation of this kinase during pain signaling. It should be noted that during normal conditions the activity and expression of Cdk5/p35 is very low in DRG, TG or nociceptor nerve endings. This suggests that molecular activation specific to nociceptor sensation may lead to Cdk5/p35 activation. In order to better understand this process, multiple labs including ours have put significant efforts in identifying the upstream activators of Cdk5/p35 during pain sensation. A systemic analysis of multiple predictors in this process revealed that tumor necrosis factor-α (TNF-α) and transforming growth factor-β (TGF-β) present in inflammatory soup and released during tissue injury, regulate Cdk5 activity, by MAPKs through subsequent activation of Egr-1 and p35 expression [33,55]. Once activated it regulates mitogen-activated protein kinase kinase 1/2 (MEK1/2) activity through a negative feedback loop [47] and ensures the constitutive activation of MAPK pathway which further maintains higher activity of Cdk5/p35 during chronic pain. This upregulated Cdk5 activity leads to phosphorylation of Cdk5 targeted substrates such as TRPV1 [42], δ-opioid receptor [56], N-methyl-D-aspartate receptor (NMDAR) NR2B subunit [37,57], P/Q type voltage-dependent calcium channel [58] and ATP-gated P2X(3) receptors [59] and regulates their function.

For some time it has been recognized that inflammatory mediators released from immune cells can contribute to the persistent pathological pain states. However, it has only recently become clear that immune cell products might have a crucial role not just in inflammatory pain, but also in neuropathic pain caused by damage to peripheral nerves or to the CNS. The immune cells have an important role as pain modulators not just in inflamed tissues, but also in damaged peripheral nerves and in the CNS. The types of immune cells that contribute to inflammatory pain depend on the inflammatory condition, however, the role of mast cells, macrophages, T and B cells have been studied well in multiple experimental pain models [60]. These immune cells can act at many anatomical levels during pain sensation such as peripheral tissues that are undergoing inflammation, peripheral nerves and the spinal cord in cases of peripheral neuropathy. During inflammation a wide range of immune mediators is released from these cells, some of which can affect pain signaling systems. We have recently identified a crucial role for Cdk5/p35 in T cell activation and inflammation associated with autoimmune disorders such as multiple sclerosis [61]. It would be interesting to further explore the activity of this kinase in other immune cells as well as its functional involvement in immune cells during inflammatory pain.

## Protein Kinases and their Plausible Interaction with Cdk5 during Pain Signaling

**Figure 2:**
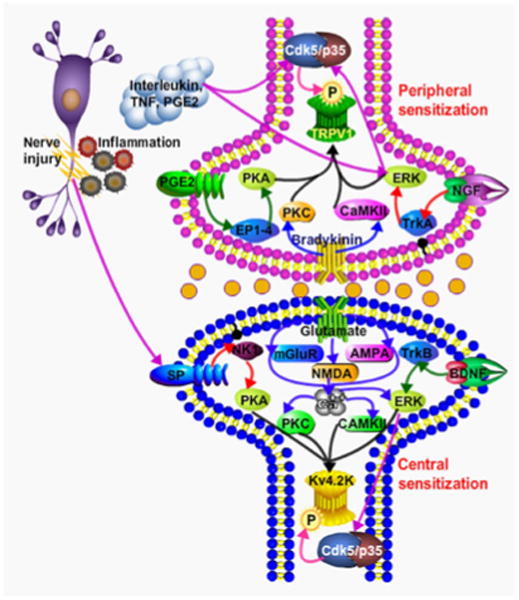
Molecular involvement of Cdk5/p35 during pain signaling: At the site of tissue injury immune cells get activated and release inflammatory soup which includes but not limited to interleukins, TNF PGE2, and NGF. These cytokines activate receptors on nociceptor terminals including TNFR, NGFR (TrkA) and PGE2 leading to the activation of other kinases such as PKA, PKC, CaMK-II, ERK. This process leads to further activation of Cdk5/p35. Activated Cdk5 leads to phosphorylation of TRPV1 and activates TRPV1 ion channel, causing peripheral sensitization. The central sensitization occurs indirectly via release of glutamate and/or neurotrophins from peripheral neurons or directly by the release of the substance P. Overall this process leads to the activation of NMDA, AMPA, mGluR and TrkB receptors by corresponding ligands and further activating downstream kinases such as PKA, PKC, CaMK-II, ERK. This process further activates the Cdk5/p35 in postsynaptic dorsal horn neurons which modulates the activity of other ion channels by their by posttranslational modification.

A growing body of literature suggests that inflammatory mediators such as prostaglandin E2, serotonin, epinephrine, and nerve growth factor (NGF) produce hyperalgesia through activation of protein kinase A or C (PKA or PKC) in primary afferent neurons [62,63]. However, it has been shown that the ERK cascade acts in epinephrine-induced hyperalgesia and the Ras-MEK-ERK pathway is activated independently of PKA or PKC [64-66]. Activation of mitogen-activated protein kinase (MAPK) in nociceptive neurons leads to pain hypersensitivity through transcription-dependent and -independent means [67,68]. Activation of these kinases may directly or indirectly increase the transcription of various immediate early genes, including *c-fos, Zif 268*, and cyclooxygenase-2 (Cox-2), as well as other early response genes, which are thought to cause a transition from short-term adaptive processes to long-term hyperexcitability of nociceptive neurons, leading to the development of chronic pain [67-74]. Therefore, it is important to understand the relationship and potential crosstalk between Cdk5 and other kinases during pain signaling (Figure 2).

## PKA, PKC, and PKG

For example, mice carrying a null mutation in neuronal-specific isoform of protein kinase A (PKA) subunit RI-β are hyporesponsive to injury-induced inflammation and pain [75]. Moreover, prostaglandin E(2) (PGE(2)), a cyclooxygenase (COX) product, is the best known lipid mediator that acts through cAMP–PKA pathway to sensitize the peripheral terminals of nociceptors during inflammatory pain [76]. The involvement of PKA in mouse model of chemical and mechanical allodynia has also been reported [77,78]. Recent data suggest that activation of spinal T-cell death-associated gene-8 (TDAG8) contributes to bone cancer pain through the PKA signaling pathway in rats [79]. Sustained morphine mediated activation of spinal cAMP/PKA dependent signaling also plays an important role in opioid induced hyperalgesia [80]. Interestingly, Cdk5 phosphorylates Thr-75 of dopamine- and cAMP-regulated phosphoprotein of 32 kDa (DARPP-32), which results in protein kinase A (PKA) inhibition [81]. This Cdk5 activity has been shown to regulate dopaminergic and glutamatergic signals, both of which are important in the molecular mechanisms of drugs of abuse including opiates [81]. Furthermore, retinoic acid treatment is shown to induce *c-fos* mediated AP-1 binding, and cAMP-responsive element binding protein (CREB) mediated CRE binding via ERK1/2 and PKA pathway, respectively, in the Cdk5 promoter region, resulting in the induction of Cdk5 [82].

The involvement of protein kinase C (PKC) in analgesia and its importance in peripheral and central sensitization during chronic pain has been discussed at length by Velázquez et al. [83]. Mainly PKCε mediates peripheral sensitization whereas PKCγ plays important role in central sensitization. By phosphorylating key intermediates PKC enhances excitatory signaling and suppresses inhibitory signaling to reduce pain threshold and induce chronic pain [84,85]. It has been shown that PKCδ stabilizes the p35 protein level by phosphorylating p35 and diminishing its ubiquitination. Furthermore, PKCδ can be activated by the brain-derived neurotrophic factor (BDNF) and is required for the activation of Cdk5 by BDNF [86]. Moreover it has been argued that PKC exerts its effects on the phosphorylation state of Cdk5 substrates through an indirect mechanism that may involve regulatory binding partners of Cdk5 other than its neuronal cofactors [87]. Mice lacking protein kinase G1 (PKG-1) show reduced inflammatory hyperalgesia with preservation of acute thermal nociception, suggesting the importance of this kinase in inflammatory pain [88].

## Calcium/calmodulin-dependent kinase-II (CaMKII)

Up-regulation of calcium/calmodulin-dependent kinase-II (CaMKII) activity in the dorsal horn of spinal cord has been reported during both neuropathic pain and central sensitization [89,90]. Recent reports further validate the involvement of this kinase in pelvic, visceral and neuropathic pain [91-93]. CaMKII has also been recognized as a potential protein kinase, which by virtue of its co-localization with μ-opioid receptor may be in a position to phosphorylate the μ-opioid receptor and may thus contribute to the development of tolerance to opioid analgesics [94]. Studies from Tsai et al. [95], have revealed that alpha-actinin-1 and the alpha-subunit of Ca^2+^/calmodulin-dependent protein kinase II (CaMKII-α), two proteins localized at the postsynaptic density; interact with Cdk5 via their association with p35 and p39. CaMKII-α and α-actinin-1 bind to distinct regions of p35 and p39 and also can interact with each other. Moreover, the association of CaMKII-α and α-actinin-1 to the Cdk5 activators, as well as to each other, is stimulated by calcium. Further, the activation of glutamate receptors increases the association of p35 and p39 with CaMKII-α, and the inhibition of CaMKII activation diminishes this effect [95]. Moreover, Cdk5 activity is known to suppress CaMKII activation, and that the down regulation of Cdk5 activity after treatment with NMDA facilitates CaMKII activation, leading to the easier induction of long-term potentiation [96].

## Tropomyosin-related kinases (Trk)

Tropomyosin-related kinases (Trks) are a family of receptor tyrosine kinases activated by neurotrophins. Trks play important roles in pain sensation as well as tumor cell growth and survival signaling. It's been shown that sustained administration of a peripherally selective Trk-A, B and C inhibitor significantly reduces skeletal pain without having any obvious detrimental effects on adult sensory and sympathetic nerve fibers [97]. Recently the potential and importance of several series of Trk inhibitors for the treatment of pain and/or cancer has been tested [98]. Neurotrophins such as NGF and BDNF enhance Cdk5 activity [99,100]. BDNF mediated activation of TrkB results in the recruitment of Cdk5 to the activated receptors, leading to phosphorylation of Cdk5 by TrkB at Tyr15 [99], which has been demonstrated to enhance Cdk5 activity [101]. This finding reveals an alternative mechanism by which neurotrophins may elevate Cdk5 activity, although whether other Trk receptors similar to phosphorylate Cdk5 remains to be explored. TrkB was also identified as a substrate of Cdk5. Inhibition of TrkB phosphorylation by Cdk5 abolishes BDNF-triggered increase in primary dendrites, in addition to attenuating BDNF-induced activation of Rho GTPase Cdc42, suggesting that phosphorylation of TrkB by Cdk5 is required for BDNF-induced dendrite growth [99]. Although the direct involvement of Cdk5 in Trk induced hyperalgesia has not been explored, it is intriguing to speculate that the inhibition of Cdk5 may abrupt neurotrophin triggered Trk signaling and thus dampen downstream nociceptive signaling cascade.

## MAPK and other kinases

Increasing evidence indicates that mitogen-activated protein kinases (MAPKs), including extracellular signal-regulated kinase (ERK), p38, and c-Jun N-terminal kinase (JNK)—are involved in inflammatory and neuropathic pain. Central mechanisms of the MAPK family of signal transduction molecules have been well defined [102]. Translation of mRNA takes place in three steps, initiation, elongation, termination, and is a rapid and reversible process spatially controlled by a large number of upstream kinases [103]. In the periphery, the necessary machinery for the mRNA translation is present in peripheral sensory axons [104]. The available evidence implies that local mRNA translation can occur in primary afferents under the control of the mTOR and ERK pathways. One form of activated mTOR is restricted to A-nociceptors and a small subset of C-fibers that signal the secondary changes in sensitivity following injury while ERK modulated local protein synthesis regulates the sensitization of C-nociceptors by inflammatory mediators [105-108]. More recently, phosphorylated forms of ERK, p38, and JNK in primary afferent neurons have been shown to produce exaggerated pain sensation, suggesting that MAPK pathways can increase pain hypersensitivity via peripheral mechanisms [109-114]. Recent pharmacological studies showed differential roles of peripheral MAPK signal transduction pathways in different types of pain induced by inflammation [115,116]. For example, inhibitors of ERK, p38, and JNK have been shown to effectively alleviate inflammatory and neuropathic pain in different animal models. However, those specific MAPK pathway inhibitors, such as PD98059 and SB203580 are very useful reagents for basic research but cannot be used as drugs for reasons of toxicity, pharmacology, or solubility. It is also surprising to find that in several preclinical studies MEK inhibitors are well tolerated, given that the ERK cascade has been implicated in many cell functions including cell growth. It also remains to be tested whether these MAPK inhibitors are beneficial for patients with different pain conditions. IKKβ-mediated NF-κB stimulation in injured primary sensory neurons promotes cytokine and chemokine production and contributes thereby to the development of chronic pain [117]. A novel and selective Rho kinase (ROCK) inhibitor AS1892802 has been shown to relieve pain symptoms in monoiodoacetate-induced arthritis and streptozotocin-induced neuropathy pain models [118,119]. Casein kinase 2 (CK2) participates in inflammatory nociception both in the acute and chronic phases and the efficacy of a novel potent substituted pyrimido[4,5-c] quinoline ATP-competitive inhibitor of CK2 has been tested for pain therapy [120,121]. The role of PI3 kinase/AKT in pain signaling has also been shown in recent reports. Genetic deletion of PI3Kγ in mice enhanced scratching behaviors in histamine-dependent and protease-activated receptor 2 (PAR-2)-dependent itch [122]. Moreover, PI3-kinase/Akt pathway has been shown to regulate membrane insertion of acid-sensing ion channel 1a (AISC1a) which underlies BDNF-induced pain hypersensitivity [123]. These findings emphasize, therefore, the importance of the mTOR and ERK pathways as a potential target for pain control. Interestingly, studies from Zheng et al. have revealed a crosstalk between Cdk5 and MAPK pathway [124,125]. Moreover, researchers have demonstrated that both the PI3K/Akt/mammalian target of rapamycin and the cdk5/p35 signal transduction pathways contribute to the induction of DARPP-32 protein levels by BDNF and that the effects are on both the transcriptional and translational levels. It also appears that PI3K is upstream of cdk5/p35, and its activation can lead to an increase in p35 protein levels [126].

## Conclusion

It is quite clear that there is an essential requirement for Cdk5/p35 signaling to mount a nociceptive response. The importance of this kinase is not only linked to pain signaling but also involved in morphine tolerance and opioid addiction, providing a dominant focus for clinical analgesic therapy. The modulation of Cdk5/p35 activity in primary afferent neurons as well as in peripheral tissues makes it an attractive potential target for novel analgesics. Usually silent in PNS, this kinase gets activated upon nociceptive stimulation by transcriptional and translational regulation of both Cdk5 and p35. Once activated, Cdk5/p35 not only interacts and regulates the activity of other protein kinases but also controls the functional regulation of multiple ion channels and other downstream signaling mediators involved in pain signaling. Past few years of research have made it clear that Cdk5/p35 provides a fundamental mechanism of nociceptive regulation in an integrated network of communication among nociceptor terminals, their peripheral target tissues, and immune cells. Therefore, blocking Cdk5/p35 activity might stop not only the initial peripheral events but also the later stages, when pain and inflammation manifests. The systemic efforts of multiple research labs involved in studying the role of Cdk5 in pain signaling put this kinase in the forefront of pain research and warrants the need of developing more specific and selective Cdk5 inhibitors and their use as pain analgesics, alone or in combination with other available pain relievers, particularly given the paucity of currently available pain medications.
